# The Ability of Microbial Community of Lake Baikal Bottom Sediments Associated with Gas Discharge to Carry Out the Transformation of Organic Matter under Thermobaric Conditions

**DOI:** 10.3389/fmicb.2016.00690

**Published:** 2016-05-10

**Authors:** Sergei V. Bukin, Olga N. Pavlova, Andrei Y. Manakov, Elena A. Kostyreva, Svetlana M. Chernitsyna, Elena V. Mamaeva, Tatyana V. Pogodaeva, Tamara I. Zemskaya

**Affiliations:** ^1^Laboratory of Hydrocarbon Microbiology, Limnological Institute, Russian Academy of ScienceIrkutsk, Russia; ^2^Laboratory of Clathrate Compounds, Nikolaev Institute of Inorganic Chemistry, Russian Academy of ScienceNovosibirsk, Russia; ^3^Laboratory of Petroleum Geochemistry, Trofimuk Institute of Petroleum Geology and Geophysics, Russian Academy of ScienceNovosibirsk, Russia; ^4^Laboratory of Hydrochemistry and Atmosphere Chemistry, Limnological Institute, Russian Academy of ScienceIrkutsk, Russia

**Keywords:** subsurface biosphere, Lake Baikal, microbial community, methane, gammacerene

## Abstract

The ability to compare the composition and metabolic potential of microbial communities inhabiting the subsurface sediment in geographically distinct locations is one of the keys to understanding the evolution and function of the subsurface biosphere. Prospective areas for study of the subsurface biosphere are the sites of hydrocarbon discharges on the bottom of the Lake Baikal rift, where ascending fluxes of gas-saturated fluids and oil from deep layers of bottom sediments seep into near-surface sediment. The samples of surface sediments collected in the area of the Posolskaya Bank methane seep were cultured for 17 months under thermobaric conditions (80°C, 5 MPa) with the addition of complementary organic substrate, and a different composition for the gas phase. After incubation, the presence of intact cells of microorganisms, organic matter transformation and the formation of oil biomarkers was confirmed in the samples, with the addition of Baikal diatom alga *Synedra acus* detritus, and gas mixture CH_4_:H_2_:CO_2_. Taxonomic assignment of the 16S rRNA sequence data indicates that the predominant sequences in the enrichment were *Sphingomonas* (55.3%), *Solirubrobacter* (27.5%) and *Arthrobacter* (16.6%). At the same time, in heat-killed sediment and in sediment without any additional substrates, which were cultivated in a CH_4_ atmosphere, no geochemical changes were detected, nor the presence of intact cells and 16S rRNA sequences of *Bacteria* and *Archaea*. This data may suggest that the decomposition of organic matter under culturing conditions could be performed by microorganisms from low-temperature sediment layers. One possible explanation of this phenomenon is migration of the representatives of the deep thermophilic community through fault zones in the near surface sediment layers, together with gas-bearing fluids.

## Introduction

The dark energy biosphere was hypothesized as a suite of habitats or ecosystems that are physically located in environments that exist in permanent darkness, and was proposed in the late 20th century (Gold, 1992). The first data on microbial activity, the total number and diversity of cultured microorganisms in the deep layers of marine sediments and continental crust (Sinclair and Ghiorse, 1989; Cragg et al., 1990; Parkes et al., 1990, 1994; Kieft et al., 1995), obtained by deep drilling, indicated that subsurface biosphere ecosystems are one of the largest microbiological habitats on Earth. To date, *Bacteria* and *Archaea* have been identified in ocean sediments at depths of up to 2500 m below sea floor (Roussel et al., 2008; Ciobanu et al., 2014; Inagaki et al., 2015). The direct measurement of cell activity indicates that the microorganisms are metabolically active in deep sediments aged >16 million years (Schippers et al., 2005).

The areas with the greatest abundance and activity of microorganisms in the subsurface biosphere are associated with high concentrations of organic matter and/or the inorganic electron donors and acceptors (D’Hondt et al., 2004; Parkes et al., 2014). An example of such “hotspots” is the tectonically active areas, where the flows of gasses and fluids ascending from deep sediments or the basaltic layer of the Earth’s crust determine the additional supply of carbon and energy (Cowen et al., 2003; Parkes et al., 2005; Engelen et al., 2008; Boetius and Wenzhöfer, 2013). Fluid flows, rich in H_2_, acetate, and methane, as well as other hydrocarbons formed in the deep sediments as a result of thermogenic activation and the degradation of kerogen (Horsfield et al., 2006; Parkes et al., 2007), may transport the representatives of the deep thermophilic community, like submarine hydrothermal vents (Hubert et al., 2009).

In addition to marine ecosystems, rift lakes, in particular Lake Baikal in Russia, are the prospective places for studying the subsurface biosphere (Biddle et al., 2012). Lake Baikal is the oldest (more than 25 million years) and deepest (maximum depth 1642 m) freshwater lake in the world. Located in a tectonically active zone, the lake has discharges of oil and gas-bearing fluids, mud volcanoes, and gas-hydrate deposits (Kuzmin et al., 2000; Kontorovich et al., 2007; Khlystov et al., 2013). Catagenesis biomarkers in the discharged oil and the isotopic composition of gas indicate that, in some areas of the lake, oil and gas migrate from depths of up to 7 km to the floor of the lake, where the temperature is close to 4°C (Khlystov et al., 2007; Kontorovich et al., 2007). The abnormally high concentrations of sulfates, bicarbonates, chlorides, and the ions of alkali and alkali-earth metals composing the pore waters of surface sediments in discharge areas may be due to the highly mineralized deep fluids passing through them (Granina et al., 2001). Ascending fluid flows also explain the presence, of the valves of ancient diatoms of the genus *Tertiarius* occurring in areas with calm sedimentation at depths of 300 m, in the upper layers of the bottom sediments of mud volcanoes (Bradbury et al., 1994; Klerkx et al., 2003). Similarly, in areas of Lake Baikal with deep-water discharges, the members of the deep thermophile community transported by the ascending fluid flows can enter the cold surface horizons of bottom sediments.

This study mainly aimed to ascertain possible existence of microorganisms, carrying out the transformation of organic matter under high temperatures typical for the deep horizons of the Earth’s crust, in the near-surface sediments of Lake Baikal. For this reason, we have cultured natural samples of sediments adjacent to the zone of tectonic fault under anaerobic conditions at temperature of 80°C and pressure of 5 MPa. We have also analyzed the microbial community using high-resolution pyrosequencing of the 16S rRNA gene fragments and characterized the processes of transformation of organic matter using gas chromatography mass spectrometry (GC-MS).

## Materials and Methods

### Site Description and Sampling

**FIGURE 1:**
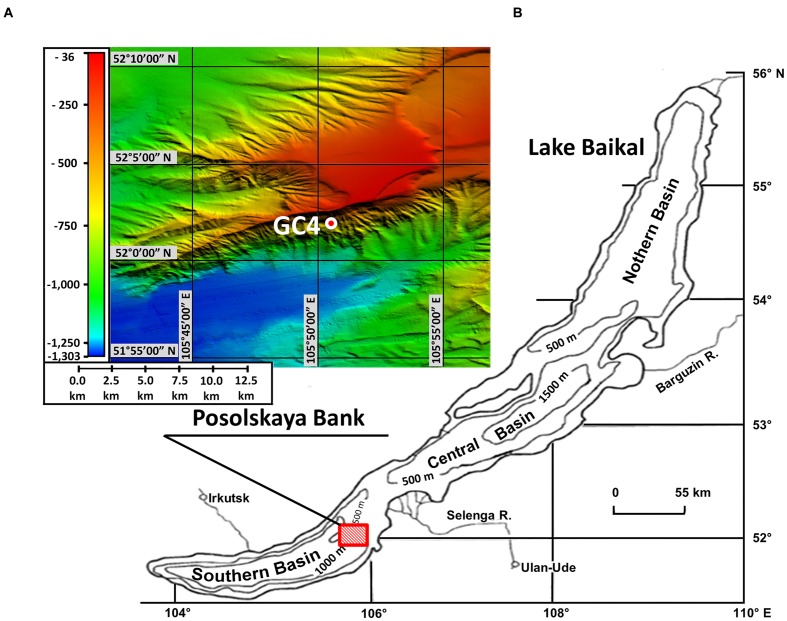
**Location of sampling area. (A)** Bathymetric map. Locations of GC4 core sample examined in this study are indicated with red-white circle. The map was created by Oleg M. Khlystov and Andrei V. Khabuev within the project No. 23.8 of the Program of RAS Presidium and the Fonds Wetenschappelijk Onderzoek project No. 1.5.198.09. **(B)** Location of underwater elevation Posolskaya Bank on the chart of Lake Baikal.

This study deals with the microbial community of bottom sediments from the Lake Baikal area of the methane seep Posolskaya Bank (Southern Basin, 52°02′10″ N, 105°50′36″ E, water depth 500 m; **Figure 1A**). The Posolskaya Bank is a pronounced underwater elevation bordering Southern and Central Basins of the lake, and is a part of the delta flank area of the Selenga River (Bezrukova et al., 2005; **Figure 1B**). The geological data (Klerkx et al., 2006; Naudts et al., 2012) as well as the analysis of the carbon isotopic composition of the discharged gas (Kalmychkov et al., 2006) show that some gas-saturated fluid flows come from deep layers below the stability zone of gas hydrates.

Bottom sediments were obtained during the expedition in June 2012 using the gravity corer (Ver-2012-06-GC4) from board of the research vessel (RV) *G.Y. Vereschagin*. After lifting the core on board, the samples of sediments for chemical analysis of pore waters, DNA extraction and experimental procedures (about 600 g) were collected. Sediment samples were taken aseptically from central part of the core with sterile 60-ml syringes (luer end removed). Chemical analysis of pore waters and DNA extraction were carried out on-board immediately after sampling. Sediments selected for the experiment from the 60–100 cm blf (cm below lake floor) layer adjoining the gas-hydrate interlayers were homogenized, divided into three parts (200 g each) and stored into liquid nitrogen (-196°C) during delivery to the laboratory.

### Chemical Analysis of Pore Water

Pore water extractions were made from 14 samples (100 g each) collected all along the core. Pore water was extracted through the 20 min sediment centrifugation (initially at 10000 rev/min and subsequently at 14000 rev/min). The anion and cation contents in water were determined according to the methods described previously (Zemskaya et al., 2010).

### Experimental Cultivation

Bottom sediments were cultured in three experimental autoclaves (Supplementary Figure S1) at 80°C and 5 MPa under different conditions of gas phase composition and type of nutrient substrate added.

In autoclave #1, the sediment sample was supplemented with detritus of *S. acus* from the pure culture obtained in a photobioreactor at the Department of Cell Ultrastructure of the Limnological Institute (Shishlyannikov et al., 2011). The culture purity was confirmed by phase-contrast and epifluorescence microscopy, plating on different media (LB, FPA/10, DA) and by PCR for amplification of a bacterial 16S rRNA gene fragment as described (Shishlyannikov et al., 2011). Diatom cells were collected on sterile polycarbonate membrane with a pore size of 1 μm (Millipore, USA), placed in sterile 1.5 ml microtubes (Axygen, USA) and stored at -80°C before experiment. The gas phase in the autoclave #1 consisted of the gas mixture CH_4_:H_2_:CO_2_ (50%:40%:10%). Autoclave #2 contained only the sediment without additional substrates and the sediments from autoclave #3 were pre-sterilized by autoclaving at 135°C for 40 min, and served as a negative control in order to confirm that microorganisms were involved in all studied processes. The gas phase in the autoclaves # 2 and #3 consisted of CH_4_.

The experiment procedure was as follows: sediment samples (200 g each) were defrosted at room temperature. Sample #1 was homogenized with 5 g of the detritus of *S. acus*. Sample (#3) was pre-sterilized by autoclaving. Further, 100 g of the each sample were stored into liquid nitrogen and then used for total organic carbon (TOC) analysis prior to the experiment; other halves of the samples (100 g) were placed in 170 ml glass beakers and saturated with sterile Baikal water in the proportion of approximately 1/4 of the sediment volume (in order to avoid drying during cultivation). The beakers were covered with Teflon lids and placed in 400 ml experimental steel autoclaves. All components were sterilized by autoclaving (135°C for 40 min). In order to avoid contamination, all handling was carried out in biosafety cabinet (BMB-II-Laminar-S-1,5, Lamsystems, Russia). The autoclaves were flushed with gas mixture CH_4_:H_2_:CO_2_ or CH_4_, increasing the pressure up to 4.2 MPa (pressure control was carried out in autoclaves integrated manometers). All gasses were sterilized by filtration. The autoclaves were placed in thermostats at a temperature 80°C where, as a result of the heating, the pressure in the autoclaves was increased up to 5 MPa.

In the autoclaves, the sediments were cultured for 17 months (the maximum period that was technically possible). At the end of the experiment, sediments from each autoclave were homogenized; 95 g of the each sample were stored into liquid nitrogen and then used for molecular genetic studies (20 g) and GC–MS analysis (75 g); 5 g of the each sample were suspended in sterile Milli-Q water and used for microscopic analysis.

### Total Organic Carbon

Content and composition of TOC were detected in the samples of bottom sediments before (considering the organic substrates added) and at the end of the experiment. Preparation of the samples, identification of the content of organic carbon (C_org_, %), isolation of soluble (bitumoid) organic matter and GC–MS analyses were performed as described previously (Pavlova et al., 2016). All measurements were triplicated.

### Microscopy

To detect the viable microorganisms in the samples, we used light and epifluorescence microscopy. For the latter, the smears prepared on glass were dehydrated in a series of solutions with rising concentrations of ethanol (50%→70%→96%, 3 min each); then they were stained with the solutions of 4′,6-diamidino-2-phenylindole (DAPI) or 3-N,3-N,6-N,6-N-tetramethylacridine-3,6-diamine (Acridine orange, AO) and observed using the microscope Axio Imager M1 (ZEISS, Germany).

### DNA Extraction

Homogenized natural sediments (10 g) and 10 g each of the three sediments samples subjected to cultivation under thermobaric conditions were used for DNA extraction. DNA was extracted according to phenol-chloroform-isoamyl alcohol method (Sambrook et al., 1989) in a modified version (Shubenkova et al., 2005). Four independent DNA extractions were carried out for each sample. In order to avoid contamination, all handling was carried out in DNA/RNA UV-cleaner box (Biosan, Latvia), using ultraviolet-treated (60 min) plasticware and micropipettes. In addition, a negative control (DNA extraction with sterile TE-buffer) was prepared for each independent DNA extractions, to ensure that no contamination with exogenous amplifiable DNA occurred during the different stages of sample treatment. Concentration and quality of extracted DNA were measured with a spectrophotometer SmartSpec Plus (Bio-Rad, USA).

### 454 Pyrosequencing and Phylogenetic Analysis

For all sediment samples, pyrosequencing was carried out using libraries of amplicons obtained by PCR with primers 341F (Muyzer et al., 1993) - 785R (Lee et al., 1993) for the V3–V4 region of the bacterial 16S rRNA gene and primers A2Fa (Reysenbach and Pace, 1995) – A519R (Sørensen and Teske, 2006) for the V1–V3 region of archaeal 16S rRNA gene. Three independent PCR mixtures were pooled for each sample to decrease PCR bias. Negative controls (reaction mixture without DNA) were included in each set of PCR reactions. PCR conditions are described in Supplementary Table S1. When direct positive PCR amplifications failed [PCR products were not visible on a 1% (w/v), agarose gel stained with ethidium bromide; for example – experimental sediment samples #2, 3; negative controls of DNA extractions], DNA extracts were additionally amplified with a nested PCR using several primer combinations (Supplementary Table S2).

Sequencing was performed using the genome sequencer GS FLX 454 (Roche, USA) with the Titanium series reagents according to the manufacturer’s recommendations. The obtained readings were analyzed using the software package Mothur 1.31.2 (Schloss et al., 2009). The sequencing errors were removed by the PyroNoise algorithm (Quince et al., 2011) selecting the sequences with the size of more than 150 bp (more than 200 bp for *Archaea*) and length of homopolymer tracts less than 6 bp which were aligned by archaeal and bacterial 16S rRNA sequences from the SILVA^1^ database. The NAST algorithm with a *k*-mer length of 8 bp was employed for sequence alignment. Chimeric sequences were identified by the UCHIME algorithm (Edgar et al., 2011) with standard parameters.

For taxonomic analysis, the obtained readings were classified by the SILVA^2^ taxonomy at a confidence threshold of 80%. The sequences with 97% similarity (genetic distance 0.03) were grouped into operational taxonomic units (OTU). Sequence clustering was based on unweighted pair group method with arithmetic mean analysis of genetic distance. Rarefaction curves, Good’s coverage, ACE and Chao1 (richness) indices and the inverse Simpson index (diversity) were calculated based on the identified OTUs using the Mothur software package. The OTU_0.03_ sequences were compared with the known 16S rRNA sequences from NCBI database using the BLASTN^3^ program with default parameters.

Nucleotide sequences of the 16S rRNA were deposited in NCBI Short Read Archive (SRAid: SRR2912888, SRR2912890, SRR2146995).

## Results

### Geochemical Characteristics of Sediments Used in the Experiment

**FIGURE 2:**
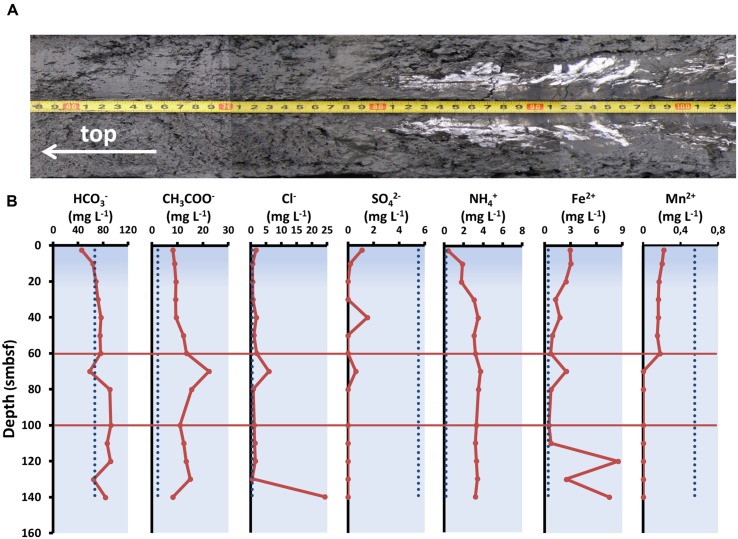
**Physico-chemical parameters of sediment used in the experiment. (A)** Structure of bottom sediment. **(B)** Concentrations of individual ions in the pore waters of bottom sediments from the core studied. The dashed lines indicate average ion concentrations in water column of Lake Baikal (according to Falkner et al., 1991; Namsaraev and Zemskaya, 2000).

The sediment layers (60–100 cm blf) used in the experiment were recovered friable gray aleuropelite silt with many cracks caused by degassing. At depths of 80 cm blf and below, there were gas hydrates forming small and massive layers (**Figure 2A**). The total number of ions in pore waters (123.06–142.83 mg l^-1^) was slightly higher than the values in sediments at the reference sites which inherited the chemical composition of the lake water column (Pogodaeva et al., 2007; **Figure 2B**). We registered high concentrations of acetate ions (up to 22.3 mg l^-1^) and ammonium ions (up to 22.3 mg l^-1^) compared to the reference sites. Concentration of sulfate ions (≤0.6 mg l^-1^) in the layer at 60–100 cm blf was insignificant in comparison with the values in upper sediment layers. Methane concentration in the studied core exceeded 90 μmol dm^-3^ of raw silt even in the upper layers of sediment (Pimenov et al., 2014).

### Phylogenetic Composition of the Microbial Community in Samples of Bottom Sediments before the Experiment

**FIGURE 3:**
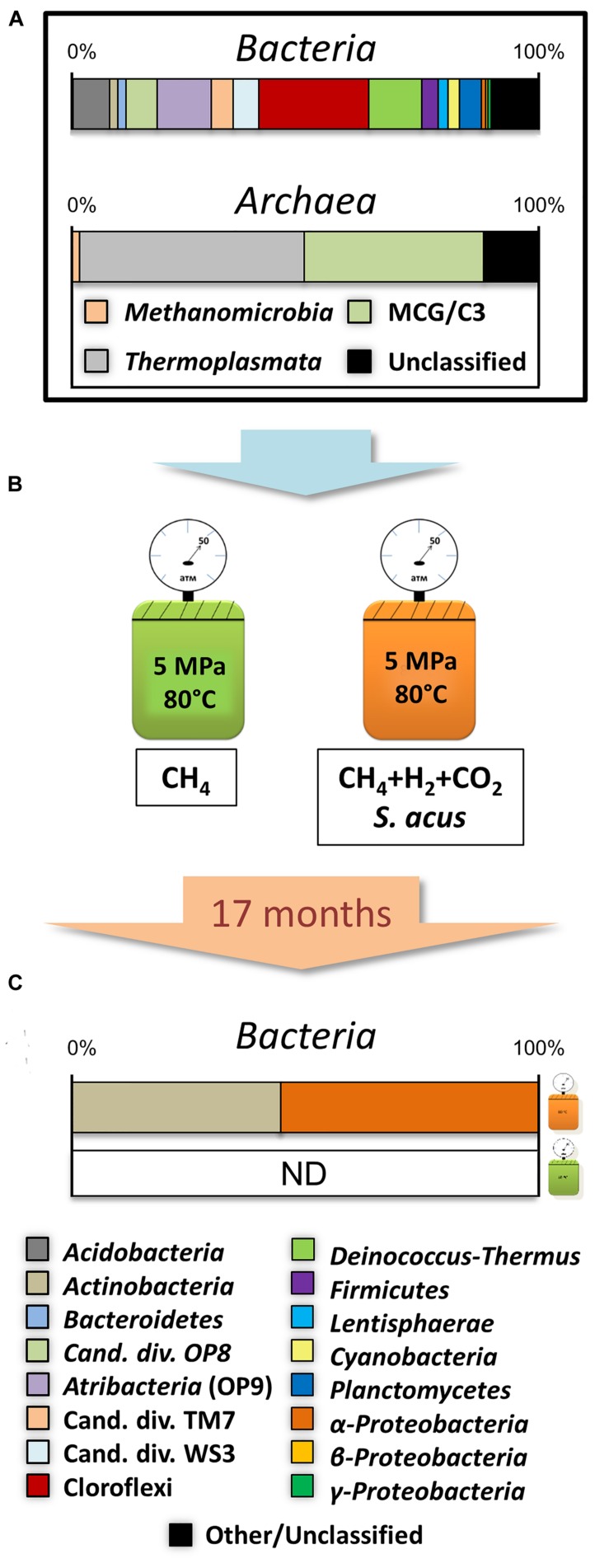
**Experimental scheme. (A)** Total composition of natural microbial community from individual layers of bottom sediments. **(B)** Culturing conditions. **(C)** Community composition of samples obtained after 17 months of culturing of bottom sediments under thermophilic conditions.

Total 16S rRNA datasets for the samples from the horizons referred to the studied layer of bottom sediments include 7093 sequences assigned to bacterial 16S rRNA gene and 4902 to the archaeal gene. Phylogenetic analysis of the communities indicated that the members of the phyla *Chloroflexi* (23.6%), candidate phylum *Atribacteria* [OP9/JS1 (Dodsworth et al., 2013; Nobu et al., 2015)] (11.6%), *Deinococcus-Thermus* (11.4%), *Acidobacteria* (7.8%), OP8 (6.7%), WS3 (5.5%), TM7 (4.8%), *Planctomycetes* (4.7%) and *Firmicutes* (3.5%) dominated *Bacteria* (**Figure 3A**). “Rare taxons” comprising less than 3% of the sequences included the phyla *Actinobacteria, Bacteroidetes, Caldiserica, Cyanobacteria, Lentisphaerae, Nitrospirae*, *Proteobacteria* (α, β, γ, and δ), *Verrucomicrobia*.

Within the *Archaea* domain, all classified sequences were distributed between the phylum *Bathyarchaeota* [Miscellaneous Crenarchaeotic Group, MCG (Evans et al., 2015)] – (32.2%) and the *Thermoplasmata* (47.9%) and *Methanomicrobia* (1.71%) classes representing the phylum *Euryarchaeota* (**Figure 3A**).

### Results of Microscopy of the Samples after Culturing

**FIGURE 4:**
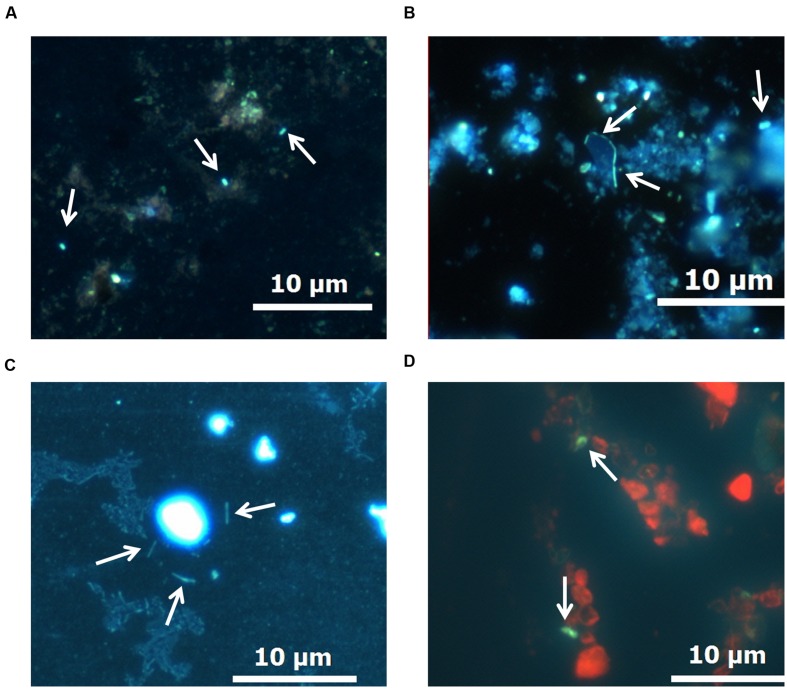
**Morphology of microorganisms in bottom sediments after the experiment. (A–C)** DAPI staining. **(D)** Acridine orange (AO) staining.

After 17 months of thermobaric culturing in the autoclaves, DAPI and AO staining indicated the single cells of microorganisms only in the samples of bottom sediments added with the detritus of the algae *S. acus*. Microorganisms associated with the sediment particles were mostly baculiform (**Figures 4A–D**).

### Results of Analysis of the Microbial Community from Bottom Sediments after 17 Months of Culturing

After the experiment, positive PCR amplifications were obtained only for DNA extracted from sample #1. A library of amplicons with primers on *Bacteria* was obtained for this sample. No positive PCR amplifications with primers on *Archaea* were obtained at that time. The DNA which was extracted from sediment samples #2 and #3 was not high-molecular, and all direct and nested PCR amplifications for these samples failed.

We obtained a library of amplification products of the 16S rRNA gene fragment comprising 13072 nucleotide sequences with an average length of 275 bp. The sequences were grouped into 41 OTU_0.03._ The rarefaction curves in Supplementary Figure S2, as well as the values of the ACE and Chao1 indices (Supplementary Table S3), indicate that the resulting sequencing volume is sufficient for the characterization of community diversity at the genus level (0.03 genetic distance).

Taxonomic classification showed that 99.4% of all sequences belonged to microorganisms of the genera *Arthrobacter* (16.6%) and *Solirubrobacter* (27.5%) associated with the phylum *Actinobacteria* (44.5%), and to the genus *Sphingomonas* (55.3%) representing the α-*Proteobacteria* class (55.3%; **Figures 3B,C**). At the same time, 0.2% of the sequences shared the phyla *Bacteroidetes*, *Cyanobacteria*, *Planctomycetes*, *Verrucomicrobia* and γ, δ- *Proteobacteria* classes, and were similar to those detected in natural sediment samples.

The comparison of the resultant sequences with the NCBI database using the BLAST protocol indicated that of the 16S rRNA sequences, the most similar to those obtained after cultivation, represented microorganisms found in the soil, water, and bottom sediments of freshwater and marine basins, as well as in groundwater and hydrocarbon contaminated ecosystems (**Table 1**).

**Table 1:** The bacteria from the GenBank database most closely related, according to 16S rRNA gene sequences identified from bottom sediments after 17 months of culturing under thermophilic conditions.

Type/class	Number of sequences	Percentage, %	Nearest 16S rRNA homolog	NCBI No.	Location	Identity, %	*E*-value
α-Proteobacteria	7224	55.3%	*Sphingomonas* sp.	LN832012	Neuston, Lake Baikal, Russia	100	2e-129
			*Sphingomonas hunanensis*	KF923436	Permafrost soils, China	100	2e-129
			*Sphingomonas* sp.	FM999997	Groundwater well, Finland	100	2e-129
			*Sphingomonas* sp.	GQ249218	Hydrocarbon-contaminated soil, China	100	2e-129
			*Sphingomonadaceae bacterium*	DQ490372	Volcanic deposits, Hawaii, USA	100	2e-129
Actinobacteria	2174	16.6%	*Arthrobacter* sp.	HQ690898	Marine sponge, South Chile Sea	100	2e-139
			*Arthrobacter* sp.	KP756664	Sea water and stone, Antarctica	99	1e-137
			*Arthrobacter agilis*	KF306343	Arctic ocean sediment	99	5e-136
			Uncultured bacterium	HQ606221	Sediment of South China Sea	99	1e-137
			*Arthrobacter* sp.	KF295504	Ice core at 78.26 m depth, China	99	5e-136
	3586	27.4%	Uncultured bacterium	JQ369448	Soil, USA	100	3e-143
			Uncultured *Actinobacterium*	EU979046	Rhizosphere soil, China	100	3e-143
			Uncultured *Solirubrobacterales* bacterium	JQ400427	Soil, USA	99	6e-140
			*Solirubrobacter* sp.^a^	FJ459990	Pine forest soil, South Korea	90	2e-95

*^a^Nearest cultured homolog*.

### Transformation of Organic Matter in the Experiment

Changes in the composition of organic matter were observed only in the sample from the autoclave #1. The TOC content of the sediment decreased during the experiment from 0.77 to 0.66%. Chloroform dissoluble bitumoid from the sediment before and after the experiment was only 0.02%; thus it was impossible to identify asphaltenes. Saturated hydrocarbons in the bitumoid before the experiment were 19.6% and asphalt-resinous compounds were 80.4%. After the experiment, the content of the latter in the sample increased to 91.2%. Before the experiment, the sample lacked aromatic fraction, whereas after the experiment it was 4.4%. Bitumoid coefficients (the ratio of the extract yield to the TOC content) in the samples before and after the experiment were 2.68 and 3.48, respectively. The ratio of acyclic isoprenoids, pristane/phytane, increased from 0.53 to 0.63. The carbon preference index (CPI), calculated according to the (1), decreased from 5.1 to 3.

(1) $$CPI=(C_{25}+C_{27}+C_{29}+C_{31})/\left(C_{26}+C_{28}+C_{30}+C_{32}\right)\,$$

The distribution curve of normal alkanes in the sample before the experiment (Supplementary Figure S3) was saw-toothed and asymmetric, with a maximum at C_31_. The n-C_27_/n–C_17_ ratio was 10. The distribution parameters of normal alkanes in the sample changed after the experiment (Supplementary Figure S3). The n-C_27_/n–C_17_ increased to 15.4.

Before and after the experiment, ethyl-cholestanes dominated the cyclic hydrocarbon biomarkers of C_27_–C_30_ steranes in the saturated fraction of the sample (37.3 and 39.2%, respectively). C_27_–C_35_ hopanes dominated terpanes (53.0% before and after the experiment). In this fraction, we also recorded an increase abundance of such biomarkers as gammacerane and retene after the experiment (Supplementary Figures S4 and S5).

## Discussion

The natural microbial community of the bottom sediments studied was represented by the taxa which are mainly widespread in freshwater ecosystems (Newton et al., 2011). However, the uncultured members of *Atribacteria* and *Chloroflexi*, which were abundant in the studied sample, are not typical of the communities of sediments in Lake Baikal (Zemskaya et al., 2015). In Baikal, as in many other freshwater lakes (Winters et al., 2014; Zhang et al., 2014; Ding et al., 2015), the most abundant phyla are *Proteobacteria* and *Actinobacteria*. Previously, communities where the taxa *Atribacteria*, *Chloroflexi*, and *Bathyarchaeota* were most abundant was identified only in the subsurface hydrate-associated sediments of the Saint Petersburg methane seep in the Central Baikal Basin (Kadnikov et al., 2011). Members of the three phyla were registered in the bottom sediments of freshwater lakes (Zhang et al., 2014; Ding et al., 2015), but they were mostly characteristic of the various specific deep marine sub-seafloor locations, including seeps and gas-hydrate sites (Orcutt et al., 2011; Parkes et al., 2014; Ruff et al., 2015). *Chloroflexi* are mainly found in bottom sediments enriched with organic matter: whereas the representatives of the candidate phylum *Atribacteria* form the basis of the communities from the hydrate-associated sediments; *Bathyarchaeota* contains a huge number of diverse phylogenetic lineages and is ubiquitous (Inagaki et al., 2006; Webster et al., 2006; Orcutt et al., 2011; Parkes et al., 2014). *Chloroflexi* and *Atribacteria* show heterotrophic metabolisms and are involved in various stages of the destruction of detritus or organic matter originating from other sources (Webster et al., 2006; Kubo et al., 2012; Dodsworth et al., 2013; Hug et al., 2013). Metabolic reconstruction of the two *Bathyarchaeota* genomes revealed the presence of genes that are diagnostic of the capacity to perform methylotrophic methanogenesis or anaerobic oxidation of methane (AOM) and also use lactate, peptides, monosaccharides, and pyruvate for energy production (Evans et al., 2015). Another interesting group of *Archaea*, which sequences were abundant in natural sediment, is *Thermoplasmata.* Although most cultured members of the *Thermoplasmata* are primarily found in oxic and/or hot environments, sequences of the uncultured representatives of this group (uncultured *Thermoplasmatales* in our case) are abundant in cold anoxic sediments (Lloyd et al., 2013). Network analysis showed that *Thermoplasmata* recurrently co-occur with *Bathyarchaeota* in sediment ecosystems, suggesting a potentially relevant trophic connection between the two clades (Fillol et al., 2015).

The abundance of *Atribacteria, Chloroflexi*, *Bathyarchaeota*, and *Thermoplasmata* in the sediments analyzed may thus be due to active processes of destruction of detrital material in the studied area of gas discharge. This correlates with the high acetate and ammonium concentrations in the core, which may be a result of both the local active anaerobic decomposition of organic matter (and/or nitrate reduction) and transportation with deep fluid flow (Krylov et al., 2008; Sass and Parkes, 2011; Boetius and Wenzhöfer, 2013). Functions of primary and secondary fermenting bacteria in the studied community can represent other abundant taxa, namely: *Acidobacteria*, *Deinococcus-Thermus*, *Firmicutes*, OP8, WS3, TM7. Representatives of *Acidobacteria* can also use iron as an electron acceptor for anaerobic respiration (Ward et al., 2009), whereas *Planctomycetes* are capable of the anaerobic oxidation of ammonium (Fuerst and Sagulenko, 2011). A small number of methanogenic *Methanomicrobia* is consistent with data suggesting that the methane in the studied area mostly comes from the underlying layers (Klerkx et al., 2006; Naudts et al., 2012).

Taxonomic assignment of the 16S rRNA sequence data indicates that the predominant sequences after cultivation were *Actinobacteria* and *Proteobacteria*, which were initially considered rare taxa. At that time, no *Archaea* were detected. Significant changes in the composition and diversity of the microbial community from natural sediments that occurred in the course of the exposure under the experimental conditions may show that the abundant taxa are likely to be indigenous to the cold near-surface bottom sediments and not able to survive at high temperature. Intact cells of microorganisms were found after experiment, however. Also, in the course of the culturing, the CPI value also decreased from 5.1 to 3, indicating that the transformation degree of the hydrocarbon component in organic matter reached 41%. Hydrocarbon molecules with an odd number of carbon atoms are known to prevail in organic matter from sediments not exposed to diagenetic and catagenetic transformations. The values of the odd/even ratio (CPI in different modifications) are therefore 5–6. During the thermal transformation of organic matter, the number of odd and even hydrocarbon molecules equalizes. The CPI value approaches one and does not change in subsequent late-catagenetic transformations (after the main stage of oil formation; Mukhopadhyay et al., 1979). In this case (culturing at 80°C), however, the observed changes probably resulted from the biological degradation processes, since these values on the one hand approach the threshold for living organisms, but on the other hand are the lower boundary of the values characteristic of metacatagenesis and ensure the transformation of organic matter free of the influence of physical factors. In nature, the temperature conditions of the thermal decomposition of organic matter (catagenesis) range from 85 to several 100°C (Schobert, 2013). So, under the experimental conditions, the microorganisms from the cold near-surface bottom sediments should thus survive at high temperature and cause deep destruction of organic matter, respiring very slowly or anaerobically.

Most of the phylotypes identified after the experiment were related to heterotrophs of the genera *Arthrobacter*, *Solirubrobacter*, and *Sphingomonas*. Bacteria of these genera are capable of utilizing a wide range of organic substrates, including aromatic hydrocarbons, chlorinated and other toxic compounds (Daane et al., 2002; Choi et al., 2004; Story et al., 2004; Nordin et al., 2005) and play an important role in the microbial communities of oil-contaminated soils (Peng et al., 2015). Cultivated strains of *Arthrobacter*, *Solirubrobacter*, and *Sphingomonas* are typical inhabitants of mesophilic soil environments, however. Furthermore, the ability to grow under anaerobic conditions due to nitrate reduction or fermentation was previously identified only in some *Arthrobacter* species (Eschbach et al., 2003). It is therefore unlikely that these microorganisms, as representatives of the indigenous microbial community of the cold near-surface bottom sediments, were able to survive under anaerobic hyperthermophilic conditions. One possible explanation for their abundance is that these microorganisms are one of the representatives of the microbial community of deep hot sediment layers which can migrate to the near surface sediment layers through fault zones, together with gas-bearing fluids. Both positive heat-flow anomalies near the venting sites and a negative heat flow anomaly zone to the south of the venting sites were registered within the Posolskaya Bank structure, which may indicate a convective fluid loop (Klerkx et al., 2006; Naudts et al., 2012). Early, some representatives of *Arthrobacter*, *Solirubrobacter*, and *Sphingomonas* were enriched from deep biosphere (Fredrickson et al., 1995; Balkwill et al., 1997; Crocker et al., 2000; Chang et al., 2007; Kobayashi et al., 2008), including thermophilic conditions (Ciobanu et al., 2014). Cultivation-independent molecular biological techniques also confirmed the presence of these bacteria in the deep biosphere (Inagaki et al., 2006; Mason et al., 2010; Breuker et al., 2011; Purkamo et al., 2013). According to one of the hypotheses, the widespread representatives of the phyla *Actinobacteria* and *Proteobacteria* may be explained by their flexible metabolism, allowing them to adapt to environmental changes that would have been maintained during the progressive burial of sediments (Ciobanu et al., 2014). In fact, many OTUs from ~40 to 60°C sediment associated with lignite coal beds at ~1.5 to 2.5 km below the seafloor in the Pacific Ocean deeper layers showed high sequence similarity to those from soil environments (*Actinobacteria*, *Proteobacteria*, and *Firmicutes*, etc.; Inagaki et al., 2015). Nevertheless, we cannot assert that representatives of *Arthrobacter*, *Solirubrobacter*, and *Sphingomonas* performed transformation of organic matter in the experiment, because they could simply have remained intact during the culturing. At that time, those thermophilic microorganisms which performed decomposition of organic matter may have lost viability as a result of exhaustion of the energy and/or carbon sources during long time cultivation. In previous studies, after culturing the microbial community of near-surface sediments of Lake Baikal under thermobaric conditions within a shorter period (11 months), thermophilic microorganisms were enriched (Pavlova et al., 2016).

The signs of microbial activity after 17 months of culturing in bottom sediments, supplemented with the *S*. *acus* detritus, can be explained by relatively high concentrations of organic matter and presence of CO_2_ and H_2_. The alga could be used as a substrate for fermenting bacteria, at that time CO_2_ could be utilized as an electron acceptor for autotrophic H_2_ utilization, such as hydrogenotrophic methanogenesis and acetogenesis. Previously, it was shown that homo-acetogenesis are activated during cultivation of bituminous coal and sand under anaerobic, CO_2_-rich conditions at 40 MPa and 40°C (Ohtomo et al., 2013). Also, authors decelerate that no methanogens were activated during their experiment (Ohtomo et al., 2013). That is consistent with the fact that no methanogenic *Archaea* were detected after our experiment. In autoclave #1 the microorganisms could also use substrates formed in the course of low-temperature activation of organic matter. It was reported that the warming of sediments during burial can activate both buried organic and inorganic compounds and stimulates both H_2_ production and prokaryotic activity, while sterile controls showed negligible H_2_ formation and other geochemical changes, even at temperatures as high as 100°C (e.g., basalt incubation for 130 days; Parkes et al., 2011).

Another distinctive feature is an increased abundance of the pentacyclic oil biomarker, gammacerane. Gammacerane is recognized as a precursor of gammacerane, a polycyclic hydrocarbon detected in sedimentary rocks dating as far back as the late Proterozoic (~850 Mya). Gammacerane is usually regarded as marking the sediment accumulation in saline environments (Sinninghe Damsté et al., 1995; Peters et al., 2005). In our case, the initial substrate was a freshwater source, as indicated by the numerical value of the isoprenoid ratios pristine/phytane. According to the data obtained in Vaz dos Santos Neto et al. (1998), gammacerane has δ^13^C values compatible with derivation from ciliates feeding on algae, cyanobacteria and another microorganisms growing under variable paleoenvironmental conditions. Gammacerane is also a major biomarker in many lacustrine oils and bitumens, including the Green River marl and oils from China, where the organic matter originated mainly from algae and bacteria (Peters et al., 2005). In our previous studies on culturing the microbial community of near-surface sediments from the cold methane seep Goloustnoe (Southern Basin), we obtained comparable data. The sediments after the experiment showed the formation of the polycyclic aromatic hydrocarbon, retene. Our experimental data confirms that retene can be generated in Lake Baikal during the process of biomass destruction of diatoms performed by a microbial community from deep bottom sediments (Pavlova et al., 2016). The experiments showed that microbial communities from bottom sediments of Lake Baikal with different chemical characteristics, but sufficient content of organic matter, under certain temperatures, are capable of destroying organic substrates followed by the formation of such biomarkers as retene and gammacerane.

## Author Contributions

TZ, OP, and SB conceived the study. OP and SB collected the samples. AM and SB carried out experimental cultivation. EK performed GC-MS analysis. TP carried out chemical analysis. SB performed all data analysis with support from EM and SC. SB wrote the manuscript with input from all authors.

## Conflict of Interest Statement

The authors declare that the research was conducted in the absence of any commercial or financial relationships that could be construed as a potential conflict of interest.

## Footnotes

1 http://www.mothur.org/wiki/Silva_reference_files

2 http://www.arb-silva.de

3 http://blast.ncbi.nlm.nih.gov
