# Supplementary material for: The Ability of Microbial Community of Lake Baikal Bottom Sediments Associated with Gas Discharge to Carry Out the Transformation of Organic Matter under Thermobaric Conditions
Source: Front Microbiol. 2016 May 10;7:690. doi: 10.3389/fmicb.2016.00690 (PMC4861714; doi:10.3389/fmicb.2016.00690)
Supplement: Supplementary file 1 [file Table_1.DOCX]

***Supplementary Material***

**Table S1**. PCR conditions and characterizations of primers

| Time and temperature conditions | 341F (Muyzer et al., 1993) and  785R (Lee et al., 1993) | A2Fa (Reysenbach and Pace, 1995) and A519R (Sørensen and Teske, 2006) |
| --- | --- | --- |
| Sequences | 341F 5′**-**CCTACGGGRSGCAGCAG-3′  785R 5′**-**CTACCAGGGTATCTAATCC-3′ | A2Fa 5′-TTCCGGTTGATCCYGCCGGA-3′  A519R 5′**-**GGTDTTACCGCGGCKGCTG-3′ |
| Initial denaturation (°C) | 96 for 2 minutes | |
| Denaturation  (°C) | 96 for 30 seconds | |
| Annealing  (°C) | 38 for 45 seconds | |
| Extending  (°C) | 72 for 40 seconds | |
| Number of cycles | 30 | |
| Final extending  (°C) | 72 for 10 minutes | |
