# Supplementary material for: The Ability of Microbial Community of Lake Baikal Bottom Sediments Associated with Gas Discharge to Carry Out the Transformation of Organic Matter under Thermobaric Conditions
Source: Front Microbiol. 2016 May 10;7:690. doi: 10.3389/fmicb.2016.00690 (PMC4861714; doi:10.3389/fmicb.2016.00690)
Supplement: Supplementary file 2 [file Table_2.DOCX]

***Supplementary Material***

**Table S2.** Primers used for the first round of nested PCR

| Primer | Target | Sequence* (5’ – 3’) |
| --- | --- | --- |
| Bac 8F | *Bacteria* 16S rRNA | AGAGTTTGATCATGGCTCAG |
| Bac 27F | *Bacteria* 16S rRNA | AGAGTTTGATCMTGGCTCAG |
| Uni 907R | *Bacteria* 16S rRNA | CCGTCAATTCMTTTGAGTTT |
| Bac 1350R | *Bacteria* 16S rRNA | CACGGGCGGTGTGTACAAG |
| A2Fa | *Archaea* 16S rRNA | TTCCGGTTGATCCYGCCGGA |
| Arch 915R | *Archaea* 16S rRNA | GTGCTCCCCCGCCAATTCCT |
| Arch 958R | *Archaea* 16S rRNA | YCCGGCGTTGAMTCCAATT |

*Degenerated primers: Y=T/C, M=A/C
