# Supplementary material for: The Ability of Microbial Community of Lake Baikal Bottom Sediments Associated with Gas Discharge to Carry Out the Transformation of Organic Matter under Thermobaric Conditions
Source: Front Microbiol. 2016 May 10;7:690. doi: 10.3389/fmicb.2016.00690 (PMC4861714; doi:10.3389/fmicb.2016.00690)
Supplement: Supplementary file 3 [file Table_3.DOCX]

***Supplementary Material***

**Table S3.** Sample coverage, species richness and species diversity indices for microbial community from bottom sediments after 17 months of culturing

| Genetic distance for  OTU clustering | Good’s coverage | Number of OTUs | ACE | Chao1 | Simpson’s Inverse Index |
| --- | --- | --- | --- | --- | --- |
| 0.01 | 0.966799 | 501 | 2807.8  (2159.3 $\div$ 3710.0) | 3040.5  (239.3 $\div$872.2) | 2.7  (2.7 $\div$2.8) |
| 0.03 | 0.997782 | 41 | 380.1  (263.5 $\div$ 558.0) | 447.0  (239.3 $\div$872.2) | 2.4  (2.4 $\div$2.4) |
| 0.05 | 0.999082 | 22 | 83.6  (52.6 $\div$ 145.9) | 55.0  (30.3 $\div$153.5) | 2.4  (2.4 $\div$2.4) |
