# Supplementary material for: The Ability of Microbial Community of Lake Baikal Bottom Sediments Associated with Gas Discharge to Carry Out the Transformation of Organic Matter under Thermobaric Conditions
Source: Front Microbiol. 2016 May 10;7:690. doi: 10.3389/fmicb.2016.00690 (PMC4861714; doi:10.3389/fmicb.2016.00690)
Supplement: Supplementary file 4 [file Image_1.PDF]

Supplementary material

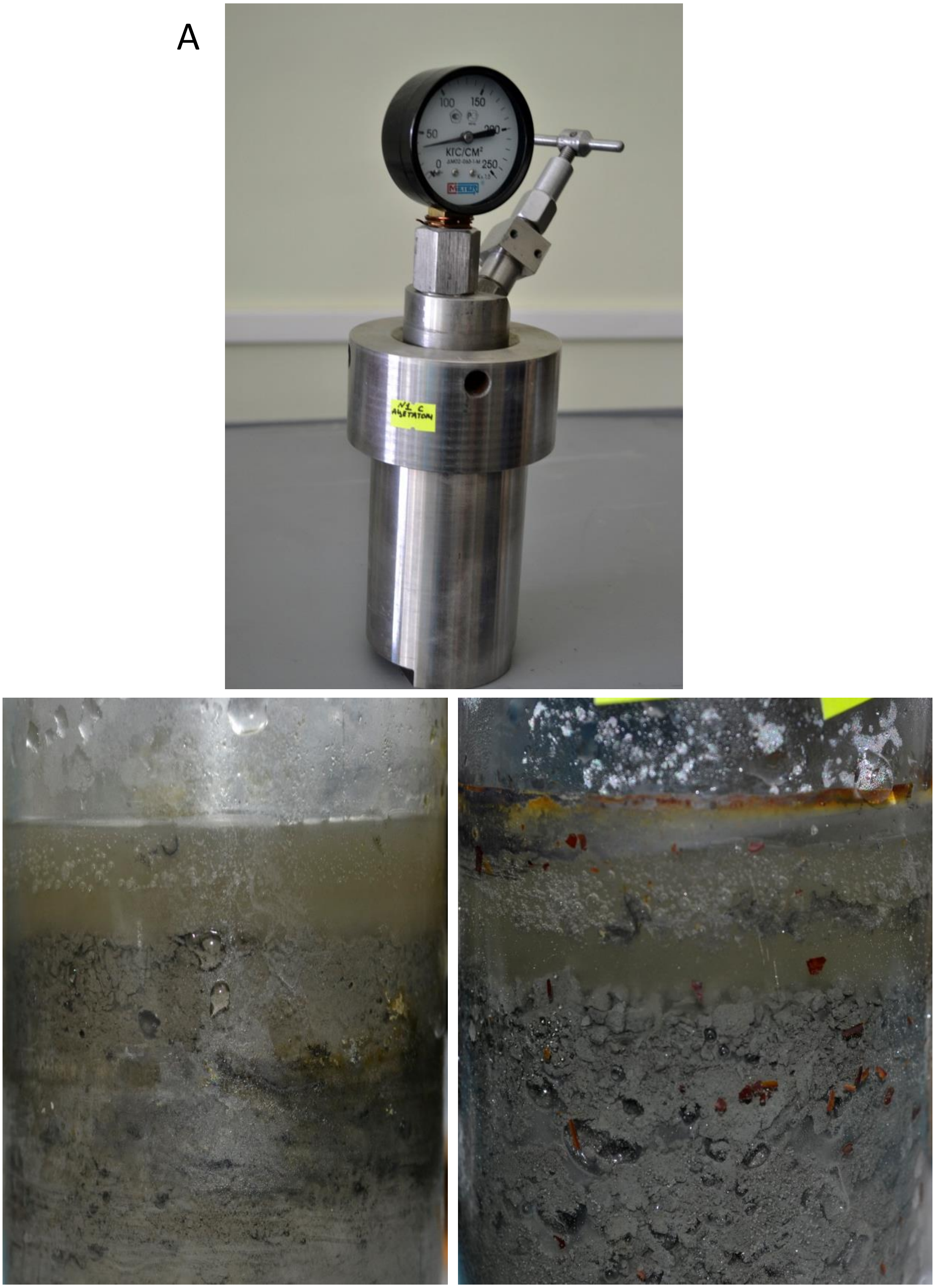

Figure S1. Experimental cultivation. (A) Experimental autoclave. **(B)** The structure of sediment without additional substrates and **(C)** sediment supplemented with detritus of *Synedra acus* after 17 months of cultivation under thermobaric conditions (80°C, 5 MPa).

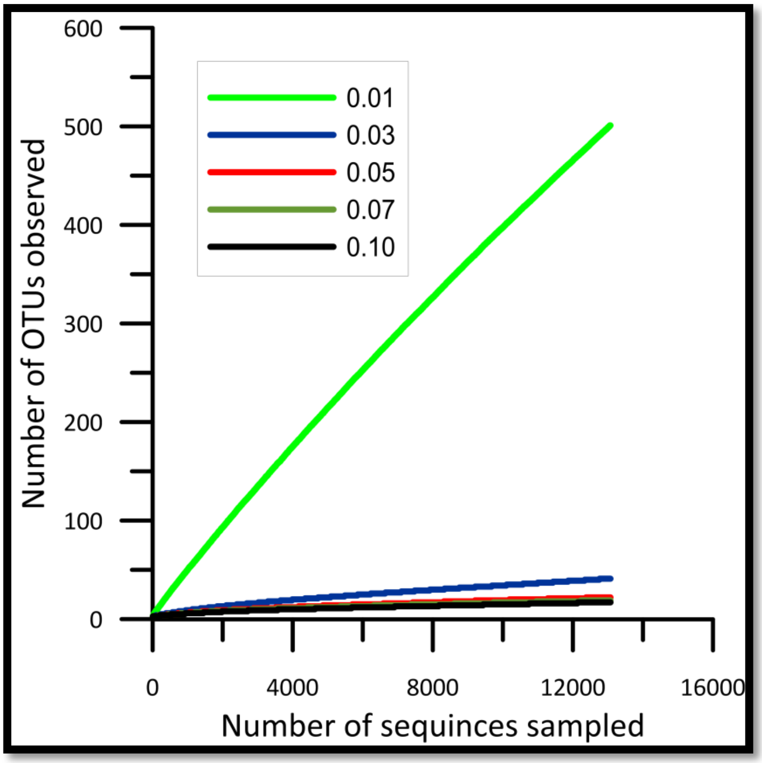

Figure S2. Bacterial diversity in samples obtained after 17 months of culturing of bottom sediments of Lake Baikal under thermophilic conditions as characterized by rarefaction curves of OTUs defined at genetic distance levels of 0.01, 0.03, 0.05, 0.07 and 0.10.

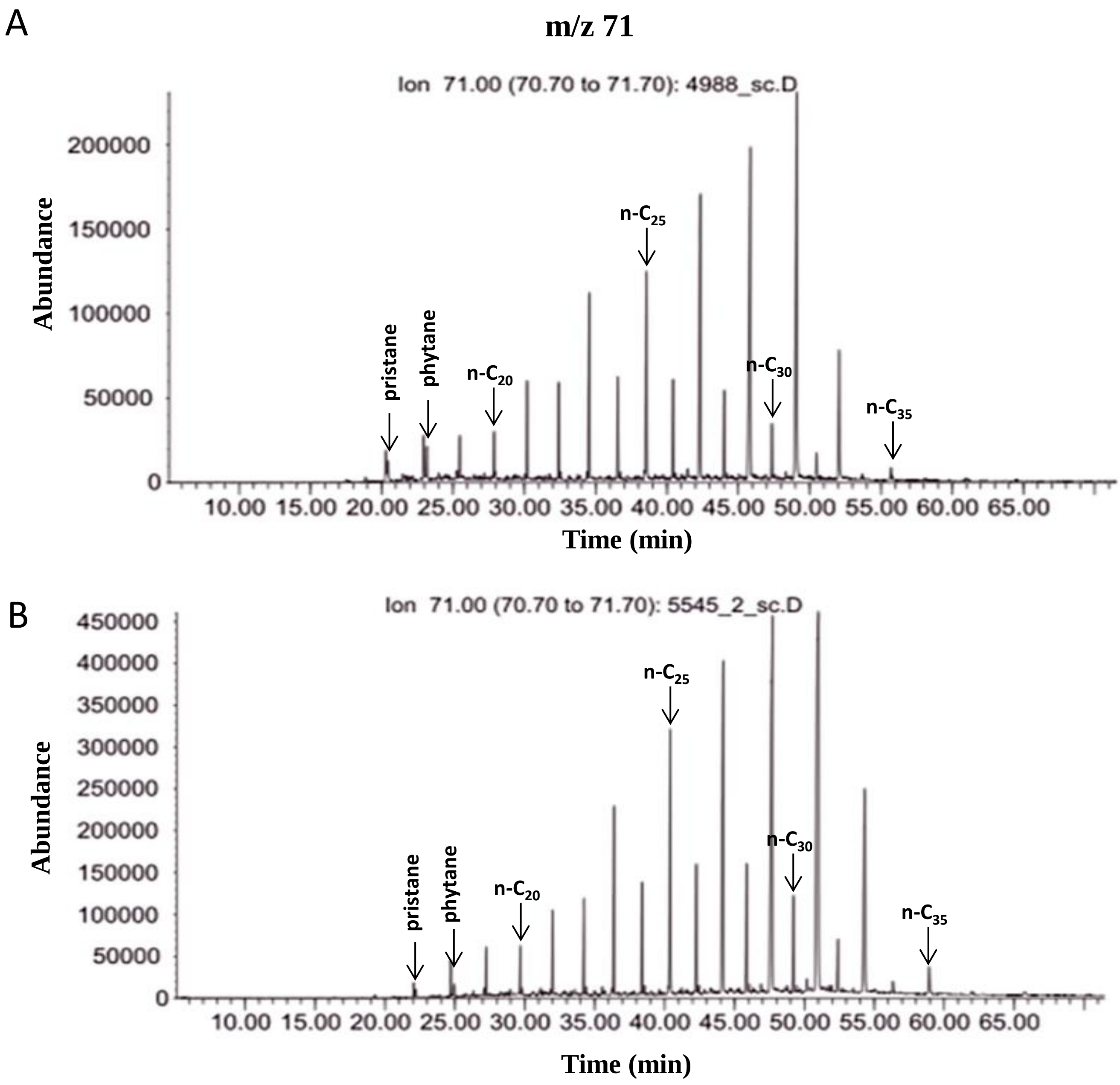

Figure S3. **Chromatography mass fragmentograms of normal alkanes and isoprenoids in the saturated fraction of bitumoids**. **(A)** Sediments before cultivation. **(B)** Sediments after cultivation.

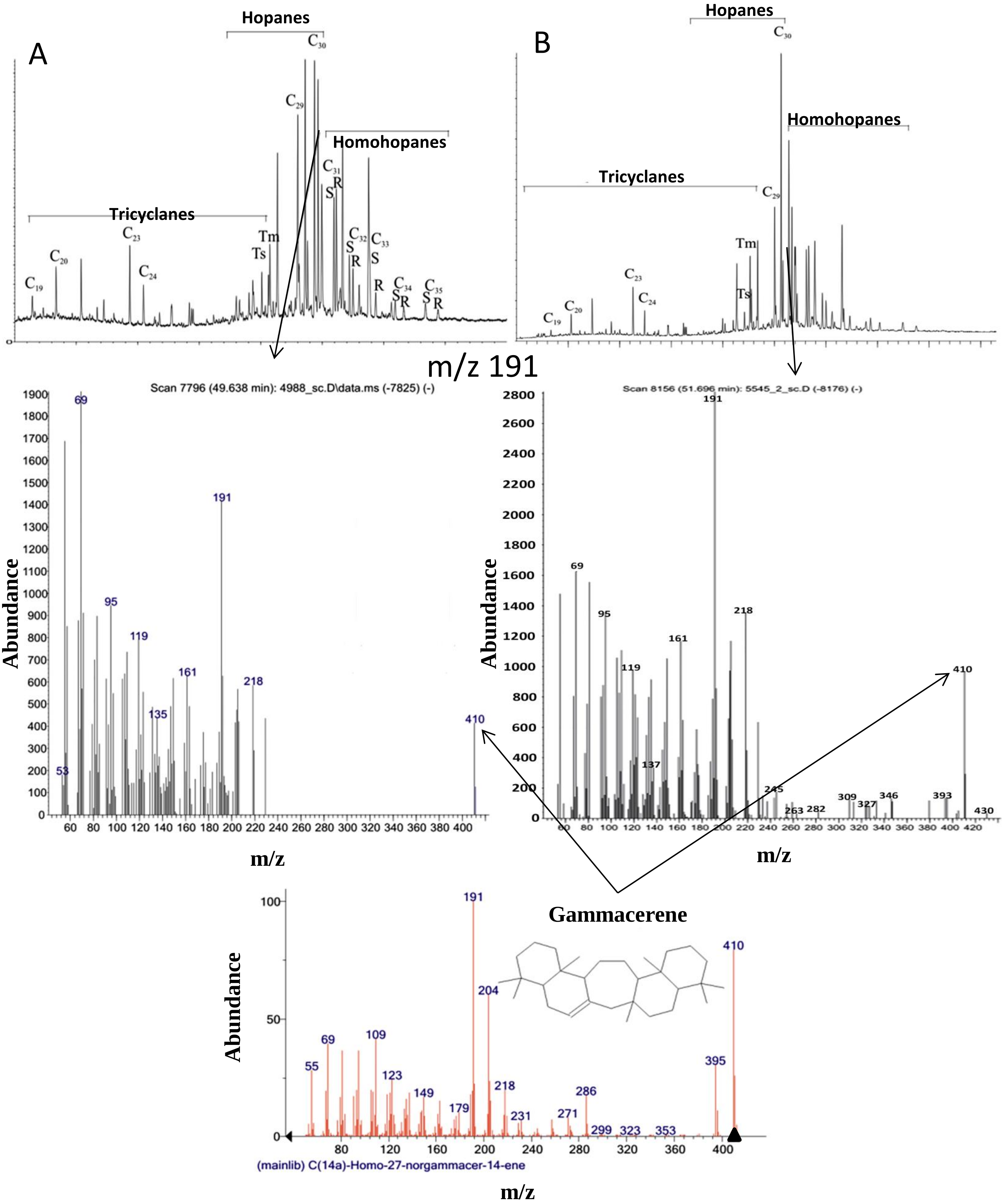

Figure S4. **Chromatography mass fragmentograms of terpanes in the saturated fraction of bitumoids (m/z 191)**. **(A)** Sediments before cultivation. **(B)** Sediments after cultivation.

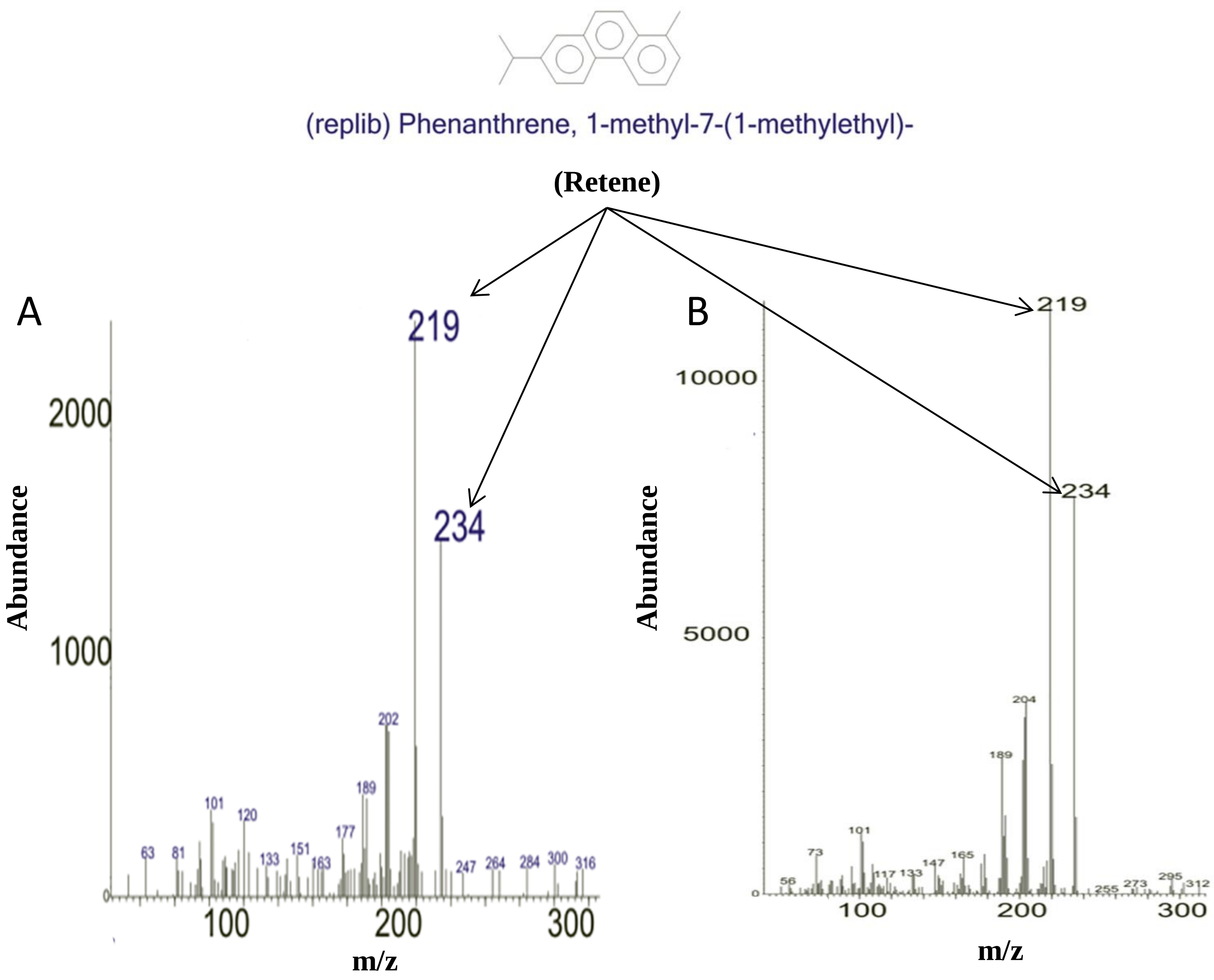

Figure S5. **Mass spectra of reten from the aromatic fraction of bitumoids**.
(A) Sediments before cultivation. **(B)** Sediments after cultivation.
